# Neoadjuvant endocrine therapy in primary breast cancer: indications and use as a research tool

**DOI:** 10.1038/sj.bjc.6605845

**Published:** 2010-08-10

**Authors:** Y H Chia, M J Ellis, C X Ma

**Affiliations:** 1 Department of Medicine, Division of Oncology, Washington University, 660 South Euclid Avenue, St Louis, MO 63110, USA; 2 Siteman Cancer Center, Washington University, 660 South Euclid Avenue, St Louis, MO 63110, USA

**Keywords:** breast cancer, oestrogen receptor, neoadjuvant endocrine therapy, genomics, relapse risk

## Abstract

Neoadjuvant endocrine therapy has been increasingly employed in clinical practice to improve surgical options for postmenopausal women with bulky hormone receptor-positive breast cancer. Recent studies indicate that tumour response in this setting may predict long-term outcome of patients on adjuvant endocrine therapy, which argues for its broader application in treating hormone receptor-positive disease. From the research perspective, neoadjuvant endocrine therapy provides a unique opportunity for studies of endocrine responsiveness and the development of novel therapeutic agents.

For patients with locally advanced breast cancer, neoadjuvant chemotherapy is commonly recommended to improve surgical outcomes. However, for postmenopausal women with oestrogen receptor (ER)-positive disease, endocrine treatment is a logical alternative because of its established efficacy in the adjuvant setting (EBCTCG, 2005) and the increasing recognition that chemotherapy may be less effective in ER+ HER2− disease (Berry *et al*, 2006; Hayes *et al*, 2007). Historically, neoadjuvant endocrine therapy was reserved for older and frail patients with ER+ breast cancer. However, recent studies of this treatment modality in younger and healthier postmenopausal women showed that the improved surgical outcomes and response observed with the endocrine approach do not show an interaction with age (Olson *et al*, 2009), justifying the increased acceptance of neoadjuvant endocrine therapy in younger postmenopausal women with better performance status. For premenopausal women, neoadjuvant endocrine therapy remains investigational. In this review, we will present results of the major neoadjuvant aromatase inhibitor (AI) trials and discuss recent progress in using neoadjuvant endocrine therapy as a research tool to assess endocrine responsiveness and evaluate novel therapeutic interventions.

## Neoadjuvant endocrine therapy: the clinical data

The potential benefit of endocrine therapy in the neoadjuvant setting was initially suggested in earlier studies of tamoxifen, as a primary treatment approach for elderly women with breast cancer who were too frail to undergo other forms of therapy such as surgery (Preece *et al*, 1982; Horobin *et al*, 1991; Bergman *et al*, 1995). The clinical response rate was in the range of 30% and higher, with long-lasting responses observed in some of these patients (Preece *et al*, 1982; Bergman *et al*, 1995). Subsequent randomised trials of tamoxifen *vs* surgery followed by tamoxifen conducted in elderly women with operable breast cancer showed that surgery is essential for optimal local control, but tamoxifen alone achieved a similar overall survival compared with surgery followed by tamoxifen (Willsher *et al*, 1997; Mustacchi *et al*, 2003; Hind *et al*, 2006; Chakrabarti *et al*, 2010). These investigations laid the foundation for the design of subsequent studies of AIs in younger and healthier postmenopausal women with bulky hormone receptor (HR)-positive disease to achieve better surgery outcome. The letrozole P024 trial (Eiermann *et al*, 2001), the Immediate Preoperative Anastrozole, Tamoxifen or Combined with Tamoxifen (IMPACT) trial (Smith *et al*, 2005) and the Preoperative ‘Arimidex’ Compared to Tamoxifen (PROACT) trial (Cataliotti *et al*, 2006) were three of these studies (Table 1).

In the P024 trial, letrozole treatment was associated with a statistically significant improvement in the rate of breast conservation compared with tamoxifen. The anastrozole-based IMPACT and PROACT trials also showed a trend favouring the AI arm, although the results in comparison with tamoxifen were not statistically significant (Smith *et al*, 2005; Cataliotti *et al*, 2006). A meta-analysis of these trials supported the notion that an AI was more effective than tamoxifen for promoting breast conservation (Seo *et al*, 2009). A promising 76% rate of breast conservation was also observed in a single arm phase II study of neoadjuvant exemestane in postmenopausal patients with HR+ tumours 3 cm or greater after 12 weeks of therapy (Mlineritsch *et al*, 2008). The American College of Surgeons Oncology Group has recently completed accrual to the randomised phase III Z1031 trial to determine whether there are any differences in efficacy between the three approved AIs in this setting (NCT00698971). Preliminary data from this trial indicate that there are no clinically significant differences between these agents as neoadjuvant treatment (Ellis *et al*, 2010).

## Preoperative endocrine therapy in premenopausal women

In an early study by Gazet *et al* (2001), 13 premenopausal women with ER+ breast cancer received neoadjuvant goserelin, a gonadotropin-releasing hormone (GnRH) analogue. At 3 months, seven of the 13 women had an overall response by clinical assessment, suggesting that premenopausal women may also benefit from neoadjuvant endocrine manipulation (Gazet *et al*, 2001). Torrisi *et al* (2007) investigated the use of letrozole and a GnRH analogue as primary therapy in premenopausal women with ER+ breast cancer. These patients received a GnRH analogue for a median of 5.2 months and letrozole for a median of 4 months. In the 32 evaluable patients, one achieved a pathological complete response (pCR) and 15 obtained a clinical and imaging partial response (Torrisi *et al*, 2007). These studies suggest that neoadjuvant endocrine therapy is effective in selected premenopausal women with ER+ breast cancer and further study in this patient population is needed.

## Comparison with preoperative chemotherapy

A direct comparison between neoadjuvant chemotherapy and endocrine therapy was reported by Semiglazov *et al* (2007), in which 239 postmenopausal women with untreated invasive breast cancers that were ER and/or progesterone (PgR) positive received either combination chemotherapy with doxorubicin and paclitaxel every 3 weeks for four cycles (*n*=118) or AI treatment with either exemestane (*n*=60) or anastrozole (*n*=61) for 3 months (Semiglazov *et al*, 2007). The clinical overall response, rates of pCR and disease progression did not differ significantly among the groups. The breast conservation rate was slightly higher in the AI groups at 33% compared with 24% in the chemotherapy arm. These findings support the hypothesis that AI therapy is an appropriate low toxicity neoadjuvant approach, but a definitive randomised study that compares neoadjuvant endocrine therapy to chemotherapy has yet to be reported. Ideally, a trial that compares the two approaches would take a predictive model forward into the clinical trial design, as subpopulations of breast cancer may benefit more from chemotherapy, while others from endocrine manipulation (van de Vijver *et al*, 2002; Paik *et al*, 2004; Parker *et al*, 2009).

More recently, the GEICAM cooperative group reported a randomised phase II trial of chemotherapy *vs* exemestane in pre- and postmenopausal women (Alba *et al*, 2010). Ninety-five patients with localised ER+, PgR+, HER2− and CK8/18+ (immunohistochemistry marker for luminal subtype) breast cancer were randomly assigned to chemotherapy (epirubicin plus cyclophosphamide every 3 weeks for four cycles followed by docetaxel every 3 weeks for four cycles) (*n*=47) or exemestane (with goserelin if premenopausal) (*n*=48). Tumour response was measured by magnetic resonance imaging. More than 50% of the participants were premenopausal women (*n*=24 in the chemotherapy arm and *n*=27 in the hormonal therapy arm). The response rate was higher for chemotherapy in the premenopausal patients (18 of the 24 in the chemotherapy arm *vs* 12 of the 27 in the hormonal therapy arm, *P*=0.027), but in postmenopausal women and those with a low baseline Ki67, responses were comparable. The underperformance of exemestane and goserelin in premenopausal women may reflect the fact that this can be inadequate for endocrine therapy in some patients, with a failure to suppress ovarian function. Alternatively, primary endocrine therapy resistance is likely to be higher in a premenopausal population and a more accurate predictive model for endocrine therapy efficacy is needed.

## Receptor status as selection criteria for neoadjuvant endocrine therapy

Oestrogen receptor positivity remains the single most important criterion for eligibility for neoadjuvant endocrine therapy. Patients with an ER Allred score of 6 and above are the most likely to respond (Ellis *et al*, 2001). The data on PgR status does not support its use as a selection criterion in the context of ER positivity, and PgR+ ER− tumours are too uncommon to make a definitive statement with regard to their management (Ellis *et al*, 2001). HER2 status has been investigated in both the IMPACT and the P024 studies. Although the presence of HER2 amplification does not preclude a meaningful response to an AI in the neoadjuvant setting (Ellis *et al*, 2001; Smith *et al*, 2005), HER2 positivity is associated with a lower suppression of Ki67, a marker of cell proliferation, in response to either tamoxifen or letrozole (Ellis *et al*, 2006), suggesting treatment resistance to endocrine therapy alone. In practice, most patients with HER2+ tumours receive trastuzumab in combination with a chemotherapy regimen because of the high pCR rate. Although the combination of trastuzumab and an AI has shown promising results in patients with metastatic disease (Marcom *et al*, 2007; Kaufman *et al*, 2009), it has not been adequately tested in the neoadjuvant setting.

## Predicting long-term outcomes after neoadjuvant therapy

**Figure 1:**
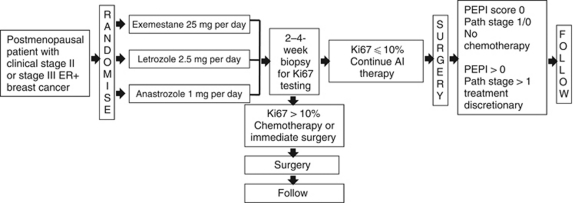
Schema for ACOSOG trial Z1031 Cohort B.

Neoadjuvant chemotherapy studies have firmly established that response, and particularly pCR, is a strong predictor of survival in breast cancer patients. However, in the subset of patients with ER+ HER2− disease treated with chemotherapy, pCR has limited value, as these patients often do well despite the absence of pCR because of effective adjuvant endocrine therapy (Buchholz *et al*, 2001). Pathological CR is very uncommon in response to neoadjuvant endocrine therapy. In three relatively large studies with letrozole, the pCR rate was no more than 1% (Eiermann *et al*, 2001; Baselga *et al*, 2009; Olson *et al*, 2009). Although residual cancer burden after neoadjuvant chemotherapy has been shown to predict distant relapse-free survival (Symmans *et al*, 2007), the application of this measure to patients who underwent neoadjuvant endocrine therapy remains to be evaluated. To identify alternative post-treatment factors that predict breast cancer survival after neoadjuvant endocrine therapy, Dowsett *et al* (2007) examined Ki67 expression before and after 2 weeks of endocrine therapy. Patients with higher Ki67 expression after 2 weeks of endocrine therapy had a significantly lower recurrence-free survival (Dowsett *et al*, 2007). In a multivariable analysis conducted on the P024 trial (Ellis *et al*, 2008a), four factors were determined to have independent prognostic value for relapse and death after relapse. These included pathological tumour size (T1/2 *vs* T3/4), pathological node status (positive or negative) and two biomarkers in the surgical resection specimen, the natural logarithm of the Ki67 value and the ER status of the tumour. A prognostic score, the preoperative endocrine prognostic index (PEPI), was developed, which weighs each of these factors according to their associated hazard ratios (Table 2). The PEPI was then validated in an independent dataset from the IMPACT trial (Ellis *et al*, 2008a). No relapses were recorded in either trial in patients with T1, N0 tumours with a PEPI score of 0 (residual tumour with Ki67 index of 2.7% – natural logarithm of 1 – or less with maintained ER expression) or in the rare patient with a pCR. These patients are not likely to benefit from adjuvant chemotherapy as endocrine therapy alone appears to adequately control their disease. These results supported an amendment to the Z1031 trial (Cohort B), in which chemotherapy was not recommended to patients in the pathological stage 1/0 PEPI 0 category. The acceptability of this advice will be assessed to see if this approach can be made a protocol mandate in the next phase of clinical trial development. In addition, patients with a high Ki67 proliferation index in a 2- to 4-week biopsy (>10%) are triaged to neoadjuvant chemotherapy or immediate surgery, as these tumours are exhibiting primary endocrine therapy resistance (Dowsett *et al*, 2007). To determine the chemotherapy responsiveness of this group, the rate of pCR with neoadjuvant chemotherapy in the high on-treatment Ki67 group will be determined (Figure 1). If this approach to tailoring neoadjuvant endocrine therapy is feasible, a larger, more definitive study will be considered.

## Genomics to determine the molecular basis for the variable response to endocrine therapy

Discovery genomics is a complex process that often requires fresh-frozen material, an adequate sample size, patient consent to genetic studies and access to sophisticated analysis techniques. To date, the number of publications on genomic profiling in the context of neoadjuvant endocrine therapy remains few and the sample sizes are small. For example, Miller *et al* (2009) reported on a series of 52 patients, 37 were considered letrozole sensitive and 15 resistant. A predictive model was developed that used all three types of gene expression variable: baseline, on-treatment and change in treatment, to differentiate between the two groups. This paper illustrates the variety of bioinformatics approaches that can be taken when before and on-treatment paired samples are available for analysis (Miller *et al*, 2009). Other investigations are ongoing to assess the predictive value of established molecular signatures, such as the Netherlands Cancer Institute NKI 70 gene (van de Vijver *et al*, 2002), 21 gene recurrence score (Paik *et al*, 2004) or the PAM50 (Parker *et al*, 2009), performed on tumour samples taken after the initiation of endocrine therapy (Ellis *et al*, 2008b). The Z1031 trial has recently completed accrual of 375 patients, despite mandatory fresh tumour tissue acquisition, through the provision of tissue acquisition kits and the active collaboration of surgical investigators. Z1031 patients are also consented for massive parallel DNA sequencing to define complete cancer genomes (Ding *et al*, 2010). Very detailed information on the molecular characteristics of endocrine therapy-resistant and -sensitive tumours is therefore a near-term prospect.

## Neoadjuvant trials to generate proof-of-principle data for novel endocrine therapy combinations

The superior efficacy of adjuvant aromatase inhibition *vs* tamoxifen in reducing the risk of recurrence was mirrored by advantages for aromatase inhibition as neoadjuvant therapy. This was most consistently seen with letrozole, with both the clinically based (Eiermann *et al*, 2001) and Ki67-based data (Ellis *et al*, 2003) correctly predicting that letrozole would be the more effective adjuvant treatment (Thurlimann *et al*, 2005) several years in advance of the actual result. Anastrozole was also shown to be a superior antiproliferative agent in comparison with tamoxifen in the neoadjuvant setting, and the failure of the anastrozole/tamoxifen combination arm to improve outcomes in the ATAC trial (Howell *et al*, 2005) was mirrored by the inferior antiproliferative response seen in the combination arm of the IMPACT trial in comparison with anastrozole monotherapy (Dowsett *et al*, 2005). These datasets provide strong arguments for the value of neoadjuvant endocrine therapy trials as a ‘proving ground’ to test the efficacy of new endocrine therapy approaches for breast cancer. Phase 2 randomised studies of endocrine therapy and signal-transduction inhibitor combinations have been recently reported for gefitinib and anastrozole (Polychronis *et al*, 2005; Smith *et al*, 2007), and the rapamycin analogue, RAD001, with letrozole (Baselga *et al*, 2009). The combination of anastrozole and gefitinib was not found to be superior to anastrozole alone. The combination of RAD001 and letrozole showed improved antiproliferative response and clinical response by palpation at the expense of more adverse events. Perhaps, the most convincing evidence for progress with a novel endocrine therapy combination would be an enhanced rate of pCR as evidence for the activation of a cell death process that would more effectively delete ER+ breast cancer cells before the acquisition of resistance to endocrine therapy (Crowder *et al*, 2009) or an increased rate of PEPI score 0 following neoadjuvant therapy.

## Conclusions

Neoadjuvant AI therapy is a clinically valuable, logical and increasingly accepted approach to neoadjuvant systemic therapy for postmenopausal women with ER+ HER2− breast cancer. With an array of new agents and predictive biomarkers to investigate, the time for further randomised trials to compare neoadjuvant endocrine treatment with conventional chemotherapy in a broad spectrum of patients with ER+ breast cancer has probably passed – particularly if the trial under consideration is not prospectively testing a predictive biomarker for endocrine responsiveness. For future investigations, we should also focus on the fact that for ER+ endocrine therapy-resistant disease, conventional chemotherapy may not be adequate treatment. We should therefore use the neoadjuvant setting to address two key issues: the development of more effective mechanism-based treatments for endocrine therapy-resistant disease; and the identification of ER+ breast cancers that can be managed without chemotherapy.

## Figures and Tables

**Table 1:** Summary of the letrozole P024, IMPACT and PROACT trials

	**Letrozole P024**	**IMPACT**	**PROACT**
	**Postmenopausal women with HR+ breast cancer**		
	**337 randomised**	**330 randomised**	**451 randomised**
Patient characteristics at baseline	None were BCS candidates at baseline; 14% deemed inoperable	Pretreatment surgical assessment available for 220 patients – 96 eligible for BCS	386 of the patients either required a mastectomy or were deemed inoperable at baseline
Definition of HR positivity	ER/PgR staining >10%	ER staining >1%	‘ER+/PgR+’
Neoadjuvant endocrine therapy	L for 4 months T for 4 months	A for 12 weeks A+T for 12 weeks T for 12 weeks	A for 3 months T for 3 months
Concomitant chemotherapy?	No	—	Yes
Primary end point	Clinical response by palpation	Overall response by caliper measurements	Overall response by ultrasound measurements
Response (per primary end point)	55% (L) *vs* 36% (T); *P*<0.001	37% (A) *vs* 39% (A+T) *vs* 36% (T)	39.5% (A) *vs* 35.4% (T)
Rate of down staging to BCS	45% (L) *vs* 35% (T); *P*=0.022	44%(A) *vs* 24% (A+T) *vs* 31% (T)	43.0% (A) *vs* 30.8% (T) in improved feasible surgery in hormone therapy-only group (*n*=314)

Abbreviations: A=anastrozole 1 mg daily; BCS=breast conserving surgery; ER=oestrogen receptor; HR=hormone receptor; IMPACT=Immediate Preoperative Anastrozole, Tamoxifen or Combined with Tamoxifen; L=letrozole 2.5 mg daily; PROACT=Preoperative ‘Arimidex’ Compared to Tamoxifen; PgR=progesterone receptor; T=tamoxifen 20 mg daily.

**Table 2:** The PEPI^a^

	**RFS**		**BCSS**	
**Pathology, biomarker status**	**HR**	**Points**	**HR**	**Points**
*Tumour size*				
T1/2	—	0	—	0
T3/4	2.8	3	4.4	3
				
*Node status*				
Negative	—	0	—	0
Positive	3.2	3	3.9	3
				
*Ki67 level*				
0–2.7% (0–1^b^)	—	0	—	0
>2.7–7.3% (1–2^b^)	1.3	1	1.4	1
>7.3–19.7% (2–3^b^)	1.7	1	2.0	2
>19.7–53.1% (3–4^b^)	2.2	2	2.7	3
>53.1% (>4^b^)	2.9	3	3.8	3
				
*ER status, Allred score*				
0–2	2.8	3	7.0	3
3–8	—	0	—	0

Abbreviations: PEPI=preoperative endocrine prognostic index; RS=relapse-free survival; BCSS=breast cancer-specific survival; HR=hazard ratio; ER=estrogen receptor.

a To obtain the PEPI score, risk points for RFS and BCSS were assigned depending on the HR defined in the P024 analysis (Ellis *et al*, 2008a). The total PEPI score assigned to each patient is the sum of the risk points derived from the pT stage, pN stage, Ki67 level and ER status of the surgical specimen. An HR in the range of 1–2 receives one risk point; an HR in the 2–2.5 range, two risk points; an HR greater than 2.5, three risk points. The total risk point score for each patient is the sum of all the risk points accumulated from the four factors in the model. For example, a patient with a T1 N0 tumour, a Ki67 staining percentage of 1% and an ER Allred score of 6 will have no risk points assigned. In contrast, a patient with a T3 N1 tumour, a Ki67 staining percentage of 25% and an ER Allred score of 2 will have a total relapse score of 3+3+2+3=11.

b The natural logarithm interval corresponding to the per cent Ki67 values on the original percentage scale.
